# Gaze Allocation and Performance Across Task-Demand Conditions During Squat-Based Exergaming: Pilot Study Using Eye Tracking

**DOI:** 10.2196/81667

**Published:** 2026-06-23

**Authors:** Keiichi Takei

**Affiliations:** 1 Department of Physical Therapy Faculty of Rehabilitation Tokyo Professional University of Health Sciences Koto-ku, Tokyo Japan

**Keywords:** exergame, eye tracking, gaze allocation, dual task, cognitive load

## Abstract

**Background:**

Exergames integrate motor, cognitive, and postural-control demands; however, how specific task-demand manipulations influence gaze allocation during exergame performance remains insufficiently characterized.

**Objective:**

This exploratory pilot study used a within-participant design to examine whether gaze allocation and task performance differed across baseline, concurrent cognitive-task, and unilateral-squat conditions during squat-based exergaming in healthy young adults.

**Methods:**

Eight healthy adults (mean age 20, SD 1 years; 7 men and 1 woman) used a squat-based exergame (*Ring Fit Adventure*; Nintendo) under 3 randomized conditions: baseline, concurrent cognitive task, and unilateral squats. In the concurrent cognitive task condition, participants performed serial subtraction during squatting. In the unilateral-squat condition, participants performed single-leg squats, which were intended to increase support-leg muscular demand as well as postural and motor-control requirements. Execution time, squat score, arithmetic performance, and eye-tracking metrics were recorded. Primary gaze outcomes were the proportion of fixation time and fixation counts allocated to predefined areas of interest (AOIs; command, avatar, and score) and to regions outside these areas across the entire trial. Differences from the baseline condition were examined using Dunnett tests or paired Wilcoxon signed-rank tests with Holm adjustment, and effect sizes were reported.

**Results:**

Exergame execution time increased from 31.0 (SD 3.3) seconds in the baseline condition to 38.2 (SD 6.1) seconds in the concurrent cognitive task condition and to 34.3 (SD 6.0) seconds in the unilateral-squat condition. In contrast, the squat score decreased from 98.8 (SD 2.8) in the baseline condition to 69.6 (SD 18.3) in the unilateral-squat condition. For fixation counts, allocation to the command AOI decreased from 44.8% (SD 11.4%) in the baseline condition to 32.1% (SD 8.9%) in the concurrent cognitive task condition and 36.2% (SD 8.5%) in the unilateral-squat condition. Outside AOI fixation counts increased in the concurrent cognitive task condition (55.7%, SD 8.6%) relative to the baseline condition (36%, SD 16.9%).

**Conclusions:**

In this exploratory within-participant pilot study, adding a concurrent cognitive task and performing unilateral squats resulted in different patterns of performance change and fixation count redistribution during squat-based exergaming. These preliminary findings suggest that AOI-based gaze allocation metrics may help characterize task demand–related attentional shifts in this setting. Larger confirmatory studies with more diverse samples and individual-level validation are needed before these metrics can be considered for adaptive or clinical applications.

## Introduction

### Background

Some studies suggest that certain video game experiences may enhance attention, memory, and executive function [1,2]. Exergames inherently involve dual-task demands because they require simultaneous motor execution and visually guided cognitive processing [3-6]. Evidence from systematic reviews, meta-analyses, and controlled studies suggests that exergame-based dual-task training can improve balance, executive function, and dual-task performance in older adults [7-10]. However, the cognitive demands imposed by exergames can vary substantially depending on specific game features, such as feedback density, pacing, and concurrent cognitive tasks, as well as individual user characteristics. Without adequately characterizing these demands, it is difficult to interpret performance changes or to adjust task difficulty rationally. Quantifying in-game cognitive load may therefore help clarify how specific task manipulations influence attentional allocation during gameplay.

Despite its importance, the internal cognitive load imposed by specific exergame task configurations remains poorly characterized [11]. Most previous studies have relied on performance metrics or subjective workload measures [12,13], which do not directly capture moment-to-moment attentional allocation during gameplay [14]. In serious game and exergaming research, cognitive load has been assessed using complementary approaches, including subjective workload ratings, performance-based indices (eg, dual-task costs and in-game accuracy or speed), and physiological measures (eg, heart rate, pupillometry, and electroencephalogram or functional near-infrared spectroscopy). However, each approach has practical limitations in dynamic whole-body tasks, including limited temporal resolution, reliance on outcome-level inference, and susceptibility to motion artifacts. Eye tracking offers a behavioral marker of moment-to-moment overt visual attentional allocation and may help characterize how specific game manipulations redistribute visual attention across task-relevant display elements.

In dual-task paradigms [15], cognitive load is often inferred from performance decrements such as slower execution time or reduced motor accuracy [16]. Although informative, these measures reflect outcome-level interference rather than the underlying dynamics of attention [17]. Objective real-time indicators of cognitive load during complex motor tasks remain an active area of investigation [18-21].

Eye tracking has increasingly been used to assess attentional allocation under cognitive load in dynamic contexts such as driving [22-24], walking [19,21], and real-world task performance [20,25]. Previous studies have reported load-dependent changes in fixation duration [20], gaze dispersion [26], and the allocation of gaze to task-relevant versus irrelevant stimuli [25]. However, findings have been inconsistent: some studies report longer fixation durations under higher cognitive load, whereas others report reduced fixation time and greater gaze variability [20,27]. In addition, relatively few studies have examined gaze allocation during whole-body exercise tasks or in exergaming contexts [21,28]. Thus, it remains unclear whether different task demand manipulations within an exergame, such as adding a concurrent cognitive task or increasing unilateral motor and postural-control requirements, are associated with distinct and measurable patterns of gaze redistribution across task-relevant display elements.

### Objectives

This pilot study aimed to examine whether area of interest (AOI)–specific gaze allocation differs across baseline, concurrent cognitive-task, and unilateral-squat conditions within a squat-based exergame. The study was not designed to isolate purely cognitive versus purely physical load. Rather, it was intended to explore whether AOI-based gaze allocation is sensitive to different task-demand manipulations within a single exergame context. By characterizing condition-dependent patterns of gaze redistribution in healthy young adults, this study sought to provide preliminary effect estimates and hypothesis-generating evidence to inform future confirmatory studies.

### Hypotheses

It was expected that the concurrent cognitive-task condition would be associated with redistribution of gaze away from task-relevant AOIs and/or increased viewing outside predefined AOIs. In contrast, the unilateral-squat condition was expected to be associated with altered sampling of task-relevant feedback, particularly the score AOI, because single-leg squatting may increase support-leg muscular demand as well as postural- and motor-control requirements. Given the exploratory pilot design, these hypotheses were directional rather than confirmatory.

## Methods

### Participants

This study was designed as an exploratory pilot study to obtain preliminary effect size estimates rather than to test hypotheses definitively. Eight young adults (mean age 20, SD 1 years; 7 men and 1 woman) were recruited from Tokyo Professional University of Health Sciences. Participants were healthy university students with no self-reported history of neurological, vestibular, or musculoskeletal disorders that could affect balance or squat performance and no known cognitive impairment. On the testing day, participants confirmed that they were free from acute illness, pain, dizziness, or excessive fatigue that could substantially affect task performance. To ensure eye-tracking data quality, contact lens users were excluded. Participants who usually wore eyeglasses performed the task without glasses because of eye-tracking requirements; those unable to clearly view the display under this condition were excluded.

### Ethical Considerations

This study was approved by the Ethics Committee of Tokyo Professional University of Health Sciences (TPU-25-002). All participants provided written informed consent before participation. No direct personal identifiers, such as names or contact information, were included in the study dataset. Participants were assigned study identification numbers, and all study data were managed and analyzed using these numbers. Only aggregate data are reported in this manuscript. Each participant received a gift card valued at ¥1000 (US $6.24 as of June 15, 2026) as compensation for participation. The study was conducted in accordance with the ethical principles of the Declaration of Helsinki.

### Experimental Protocol

**Figure 1:**
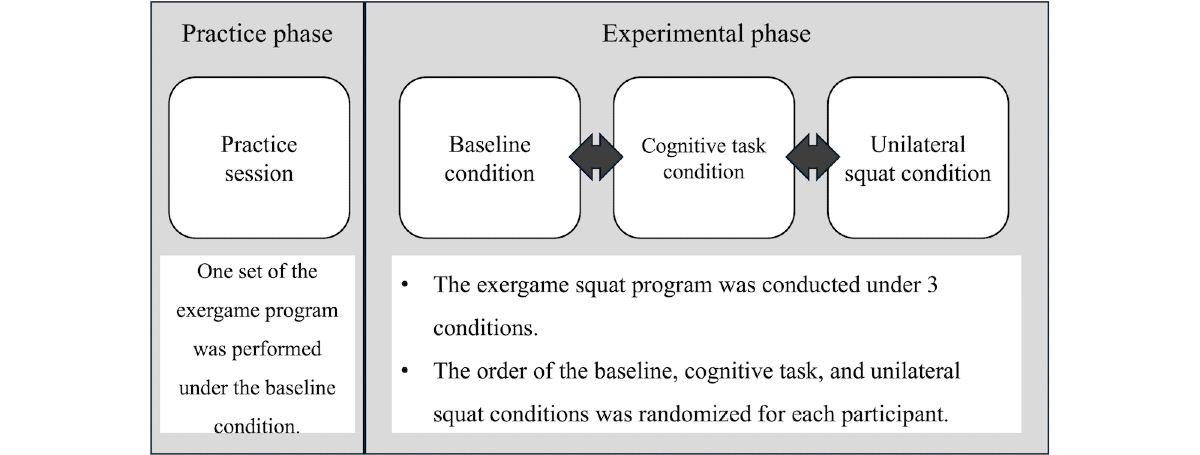
Study protocol. Baseline condition: exergame performed in a bilateral stance without an additional task; cognitive task condition: exergame performed with serial subtraction during squatting; and unilateral squat condition: exergame performed in a single-leg stance.

The experimental procedure is shown in Figure 1. The study used a repeated measures design with condition as the within-participant factor. Participants first completed 1 familiarization set under the baseline condition to become accustomed to the exergame task and interface. After familiarization, they performed 1 experimental set under each of 3 conditions: baseline, concurrent cognitive-task, and unilateral-squat conditions. The order of the 3 experimental conditions was randomized using a counterbalanced schedule, rather than administering the baseline condition first, to minimize potential order effects, including practice or familiarization effects, fatigue, and expectancy, that could systematically bias condition comparisons. A seated rest period of 3 minutes was provided between conditions to reduce immediate fatigue and potential carryover effects from the preceding condition. This interval was selected because each exergame set was brief, consisting of 10 squat repetitions, and was consistent with the use of short rest intervals in previous exergame-based rehabilitation protocols involving squat and single-leg balance tasks [29].

Across all conditions, squat execution was standardized. Participants held the Ring-Con (Nintendo) with both hands in front of the chest throughout the task. Stance width was set at approximately shoulder width. Squat tempo was guided by in-game visual cues (“lower,” “keep,” and “return”), resulting in a constant externally paced movement rhythm. Participants were instructed to aim for a squat depth corresponding to approximately 90° of knee flexion.

In the concurrent cognitive-task condition, participants performed a mental arithmetic task requiring serial subtraction of 7 from a 2-digit starting number [30,31]. For each set, a different starting number (range 70-99) was selected from a prepared list (eg, 83 and 76). Participants responded aloud in a self-paced manner as quickly and continuously as possible during squatting. Task prioritization followed a motor-first approach: participants were instructed to prioritize squat execution while continuing the arithmetic task as much as possible without compromising squat performance. No feedback on response accuracy was provided. If a participant was unable to provide a response within 5 seconds, they were instructed to skip the current subtraction and continue with the next calculation.

In the unilateral-squat condition, participants performed the same exergame squat set (10 repetitions) in a single-leg stance. This condition was intended to increase support-leg muscular demand as well as the postural stability and motor-control requirements inherent to unilateral squatting [32,33]. The supporting leg was fixed on the left side because the leg sensor was attached to the left thigh, and this standardization ensured consistency in motion capture and scoring. The nonsupporting leg was maintained in a flexed position behind the body, and no external balance support was allowed.

### Apparatus and Exergame Program

The experimental setup is shown in Figure S1 in Multimedia Appendix 1. The exergame was conducted using *Ring Fit Adventure* for the Nintendo Switch (Nintendo) [34-37], with the squat task selected from the custom mode. The Ring-Con, a ring-shaped controller held with both hands, and a leg sensor attached to the left thigh enabled participants’ movements to be reflected by an on-screen avatar. The squat program required repeated squats in response to visual cues and provided a squat score (0-100 points) that primarily reflected squat depth and timing relative to the cues.

The squat program in the custom mode of *Ring Fit Adventure* was selected for 3 reasons. First, the user interface layout remains stable in this mode, facilitating consistent definition of AOIs for eye-tracking analysis. Second, the task structure is simple and repetitive, allowing the serial-subtraction and unilateral-squat manipulations to be implemented within the same exergame task while minimizing changes in task rules and visual content. Third, the program was set to 10 repetitions to keep each trial brief, thereby reducing eye-tracking data loss and fatigue, which was appropriate for this pilot study. With respect to difficulty settings, the custom mode allows selection of the number of squat repetitions but does not provide additional difficulty parameters. Therefore, task demands were standardized across participants by using 10 repetitions in all conditions.

The game was projected onto a 130 × 230 cm screen, with participants positioned 2.5 m away. The projection was adjusted so that the center of the display was approximately at eye level during standing. Ambient lighting conditions were standardized using indoor lighting, and window light was minimized by drawing curtains to reduce glare and fluctuations in screen luminance. To reduce extraneous visual stimuli that could influence gaze dispersion, posters and other nonessential visual materials around the screen were removed, and the area surrounding the display was kept as visually uncluttered as possible.

Eye movements were recorded using a wearable eye tracker (Tobii Pro Glasses 3, Tobii Technology; sampling rate 100 Hz), and the device was recalibrated before each test condition. Gaze data were processed offline using Tobii Pro Lab (Tobii Technology) to extract fixation metrics.

### Outcome Measures

Feasibility outcomes were recorded to characterize the practical implementation of mobile eye tracking during squat-based exergaming. These outcomes included (1) completion rate, defined as the proportion of participants who completed all 3 conditions; (2) calibration success, defined as successful eye tracker calibration before each condition; and (3) technical issues, defined as any device or software malfunction or recording failure requiring restart, repeat calibration, or trial exclusion.

The primary gaze outcomes were the proportion of fixation time and the proportion of fixation counts allocated to each predefined AOI (command, avatar, and score) and to regions outside these AOIs across the entire exergame trial. These proportions were calculated by dividing fixation time or fixation count within each AOI by the total fixation time or count recorded across the whole screen during the trial. This normalization was used to characterize relative gaze allocation while reducing potential bias from condition-dependent differences in trial duration and total fixation behavior. Accordingly, proportional AOI metrics should be interpreted as indexes of relative allocation rather than absolute gaze exposure. Fixations were defined using the velocity-threshold identification (Attention) filter based on eye angular velocity (threshold 100°/s) [38].

AOI metrics (fixation duration and fixation count) were exported for each AOI. Invalid samples due to blinks or temporary tracking loss were treated as missing by the software and were not manually interpolated. No additional data cleaning beyond the software’s standard validity handling was applied. No outlier removal (eg, winsorization or participant-level exclusion based on gaze metrics) was performed.

Three AOIs were defined in Tobii Pro Lab: the command area displaying the next movement cue, the avatar area mirroring the participant’s movement, and the score area presenting squat performance points (Figure S2 in Multimedia Appendix 1). AOI dimensions were estimated from photographed screen measurements and converted to degrees of visual angle using the viewing distance of 2.5 m. The resulting AOI sizes were approximately 9.0°×21.6° for the command AOI, 15.5°×10.7° for the avatar AOI, and 11.2°×12.2° for the score AOI.

To provide an intuitive visualization of gaze distribution, scanpaths and heat maps were generated from aggregated gaze data across participants. However, these served only as qualitative supplementary materials and were not subjected to statistical testing.

Secondary outcomes were exergame execution time (seconds) and squat score (0-100 points), recorded at the end of the squat program.

### Manipulation Check

To examine whether the concurrent cognitive task and unilateral-squat manipulations altered task performance, additional performance indexes were assessed.

For the concurrent cognitive task condition, arithmetic performance was used as an objective manipulation check of the added cognitive task. Accuracy was calculated as percentage correct, defined as the number of correct responses divided by the total number of spoken responses during the set. Scoring was performed sequentially: a response was counted as correct if it was exactly 7 less than the immediately preceding response, regardless of whether the participant had made an earlier error. As individual response time stamps were not recorded, response latencies could not be calculated. Instead, the total number of responses during the set was reported together with execution time as indirect performance-based indicators related to response timing. Squat score and execution time were also analyzed as task performance indicators during the concurrent cognitive task.

For the unilateral-squat condition, squat score and execution time were analyzed as indicators of movement performance under increased unilateral motor- and postural-control requirements. In this pilot study, direct physiological indexes of exertion (eg, heart rate, ratings of perceived exertion, and electromyography) were not collected. Accordingly, the unilateral-squat condition should not be interpreted as a directly quantified increase in physiological load or as a purely physical load manipulation. Instead, changes in execution time and squat score were used as objective performance-based markers indicating that the unilateral-squat manipulation altered task difficulty relative to baseline.

### Statistical Analysis

Normality for each outcome was assessed using the Shapiro-Wilk test. For outcomes meeting the normality assumption, a 1-factor repeated measures analysis of variance with condition as the within-participant factor was performed, followed by Dunnett multiple comparison tests using the baseline condition as the reference (concurrent cognitive task condition vs baseline condition and unilateral-squat condition vs baseline condition). For outcomes violating the normality assumption, paired Wilcoxon signed-rank tests were used to compare each condition with baseline condition, and *P* values were adjusted using the Holm procedure for the 2 planned comparisons. Descriptive statistics are presented as mean (SD). Effect sizes for comparisons with the baseline condition are reported as Hedges *g* for paired samples. For each planned within-participant comparison, Hedges *g* was calculated using complete paired observations as the standardized mean change, defined as the mean within-participant difference score divided by the SD of the corresponding difference scores. Difference scores were calculated as the task demand condition minus the baseline condition, and Hedges small-sample correction was applied. Thus, positive values indicate higher values in the task demand condition than in the baseline condition, whereas negative values indicate lower values. Individual participant data were visualized alongside summary statistics (mean and SD) to enhance transparency.

Exploratory correlation analyses were conducted to examine associations between gaze allocation metrics and performance outcomes. Given the small sample size, these analyses were considered hypothesis generating; Spearman ρ and corresponding *P* values are reported, and findings were interpreted cautiously.

All analyses were performed in R (version 4.4.2; R Foundation for Statistical Computing) [39] using the effectsize package and related statistical functions [40-42].

## Results

### Feasibility Outcomes

All participants (n=8) completed all 3 conditions. Eye tracker calibration was successful in 100% (24/24) of attempts, with calibration performed before each condition for each participant. No technical issues (eg, device or software malfunction, or recording failure) occurred, and no trials were excluded for technical reasons.

### Manipulation Check

**Figure 2:**
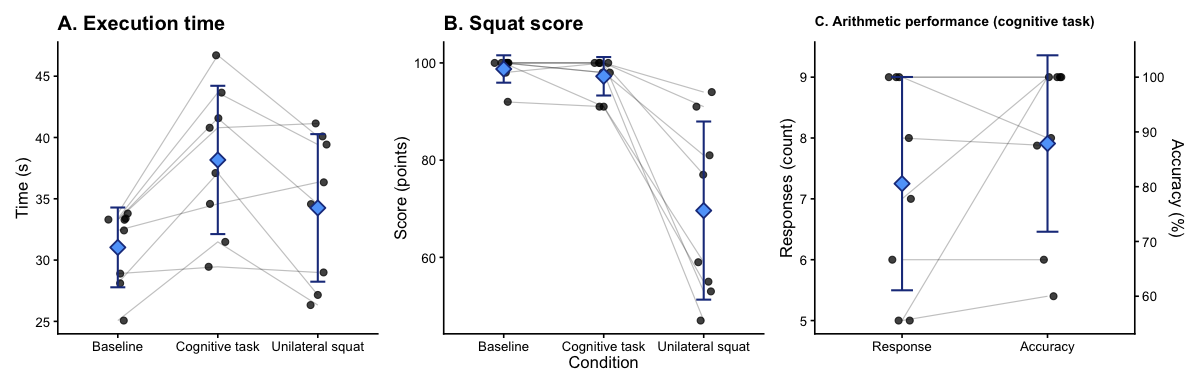
Manipulation check outcomes across conditions: (A) exergame execution time (seconds), (B) squat score (0-100 points), and (C) arithmetic task performance under the concurrent cognitive task condition. Individual participant data are shown as jittered points connected by thin lines to illustrate within-participant changes across conditions (A and B) and between arithmetic metrics (C). Diamonds indicate mean values, and error bars represent the mean+SD and mean−SD. Baseline: exergame performed in a bilateral stance without an additional task; cognitive task: exergame performed with serial subtraction during squatting; and unilateral squat: exergame performed in a single-leg stance.

Manipulation check outcomes are summarized in Table 1. As shown in Figure 2, participants performed the arithmetic task with high accuracy (mean 87.9, SD 16.1) and a consistent number of responses (mean 7.3, SD 1.8). Execution time was longer in the concurrent cognitive task condition than in the baseline condition (mean difference 7.1 seconds, 95% CI 3.7-10.5; *P*=.002), indicating measurable dual-task interference.

**Table 1:** Manipulation check outcomes for performance measures across conditions (n=8)^a^.

Outcome	Baseline, mean (SD)	Cognitive task, mean (SD)	Unilateral squat, mean (SD)	Cognitive task–baseline, mean difference (95% CI)	*P* value	Hedges *g*	Unilateral squat–baseline, mean difference (95% CI)	*P* value	Hedges *g*
Exergame execution time (s)	31.0 (3.3)	38.2 (6.1)	34.3 (6.0)	7.1 (3.7 to 10.5)	.002	1.55	3.2 (0.5 to 5.9)	.02	0.88
Squat score (0 to 100)	98.8 (2.8)	97.2 (4.0)	69.6 (18.3)	−1.5 (−4.3 to 1.3)	.23	−0.4	−29.1 (−43.9 to −14.3)	.002	−1.46
Arithmetic accuracy (%)	N/A^b^	87.9 (16.1)	N/A	—^c^	—	—	—	—	—
Arithmetic responses (n)	N/A	7.3 (1.8)	N/A	—	—	—	—	—	—

^a^Baseline: exergame performed in a bilateral stance without an additional task. Cognitive task: exergame performed with serial subtraction during squatting. Unilateral squat: exergame performed in a single-leg stance. Comparisons with baseline were performed using Dunnett test when parametric assumptions were met; otherwise, paired Wilcoxon signed-rank tests with Holm adjustment were used. Effect sizes represent Hedges *g* for paired comparisons. Arithmetic measures were collected only during cognitive task.

^b^N/A: not applicable (arithmetic outcome was not applicable because it was measured only in the cognitive task condition).

^c^No comparison with baseline was performed.

In the unilateral-squat condition, squat score was markedly lower than in the baseline condition (mean difference −29.1 points, 95% CI −43.9 to −14.3; *P*=.002), and execution time was also longer (mean difference 3.2 seconds, 95% CI 0.5-5.9; *P*=.02), although the magnitude of this increase was smaller than that observed in the concurrent cognitive task condition. Together, the substantial reduction in squat score and the smaller but significant increase in execution time suggest that the unilateral-squat condition increased movement difficulty relative to baseline.

### Gaze Allocation Across AOIs

#### Proportion of Fixation Time per AOI

**Figure 3:**
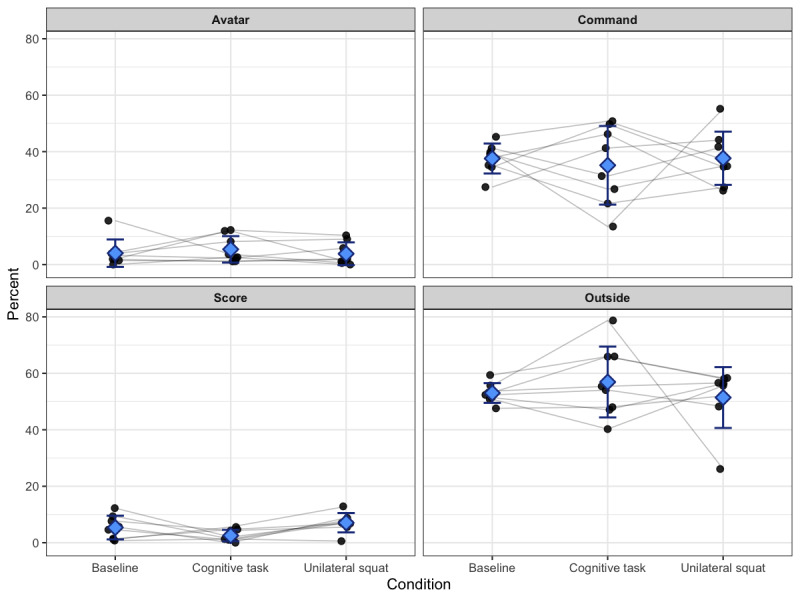
Proportion of fixation time allocated to each area of interest under baseline, concurrent cognitive task, and unilateral squat conditions. Baseline: exergame performed in a bilateral stance without an additional task; cognitive task: exergame performed with serial subtraction during squatting; and unilateral squat: exergame performed in a single-leg stance. Individual participant data are shown with mean (SD). Proportions were calculated relative to total fixation time across the entire exergame trial.

To further characterize the redistribution of visual attention under different task demand conditions, the proportion of fixation time allocated to each predefined AOI (command, avatar, and score) and to regions outside these AOIs was analyzed (Table 2; Figure 3). In the concurrent cognitive task condition, the proportion of fixation time directed outside the predefined AOIs showed a descriptive increase relative to the baseline condition, whereas the proportions allocated to the command and score AOIs showed descriptive decreases. However, these changes were modest and did not reach statistical significance. In contrast, in the unilateral-squat condition, the proportion of fixation time allocated to the score AOI showed a descriptive increase relative to the baseline condition, accompanied by a slight descriptive decrease in fixation time directed outside the predefined AOIs. The plotted data suggested greater interindividual variability in fixation time allocation in both task demand conditions than in the baseline condition.

**Table 2:** Proportion of fixation time allocated to each area of interest (AOI) across task demand conditions (n=8)^a^.

AOI	Baseline, mean (SD)	Cognitive task, mean (SD)	Unilateral squat, mean (SD)	Cognitive task minus baseline, mean difference (95% CI)	*P* value	Hedges *g*	Unilateral squat minus baseline, mean difference (95% CI)	*P* value	Hedges *g*
Command	37.6 (5.3)	35.2 (13.9)	37.7 (9.4)	−2.4 (−15.1 to 10.3)	.99	−0.14	0.1 (−8.8 to 9.0)	.99	0.01
Avatar	4.0 (4.9)	5.4 (4.7)	3.8 (4.0)	1.4 (−4.3 to 7.0)	.99	0.18	−0.2 (−6.0 to 5.6)	.99	−0.03
Score	5.4 (4.2)	2.5 (2.0)	7.1 (3.4)	−2.8 (−7.2 to 1.5)	.34	−0.48	1.7 (−2.6 to 6.1)	.38	0.29
Outside	53.0 (3.5)	56.9 (12.6)	51.4 (10.8)	3.9 (−4.8 to 12.6)	.64	0.33	−1.6 (−11.5 to 8.2)	.71	−0.12

^a^Baseline: exergame performed in a bilateral stance without an additional task. Cognitive task: exergame performed with serial subtraction during squatting. Unilateral squat: exergame performed in a single-leg stance. Values are presented as mean (SD) of the proportion (%) of fixation time within each AOI across the entire exergame trial. Proportions were calculated by dividing whole fixation duration within each AOI by the total fixation duration recorded across the entire exergame trial. Mean differences were calculated as each task demand condition minus the baseline condition. The 95% CIs were calculated from within-participant difference scores. Comparisons with baseline were performed using Dunnett test when parametric assumptions were met; otherwise, paired Wilcoxon signed-rank tests with Holm adjustment were used. Effect sizes are reported as Hedges *g* for within-participant comparisons. The “outside” category represents fixation time allocated outside the 3 predefined AOIs (command, avatar, and score).

#### Proportion of Fixation Counts per AOI

**Figure 4:**
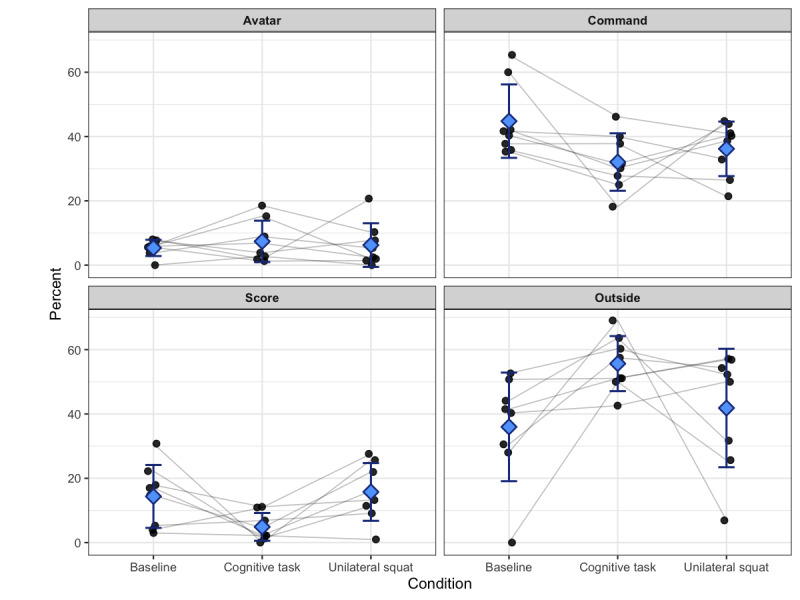
Proportion of fixation counts allocated to each area of interest (AOI) across task demand conditions. The proportion of fixation counts directed to the command, avatar, score, and outside AOIs across the entire exergame trial is shown for the baseline, concurrent cognitive task, and unilateral squat conditions. Baseline: exergame performed in a bilateral stance without an additional task; cognitive task: exergame performed with serial subtraction during squatting; and unilateral squat: exergame performed in a single-leg stance. Each proportion was calculated as the number of fixations within an AOI divided by the total number of fixations recorded during the trial.

The proportion of fixation counts allocated to each AOI varied across conditions (Table 3; Figure 4). Compared with the baseline condition, the concurrent cognitive task condition showed a lower proportion of fixation counts directed to the command AOI (mean 44.8%, SD 11.4% vs mean 32.1%, SD 8.9%; *P*=.04) and score AOI (mean 14.4%, SD 9.8% vs mean 4.9%, SD 4.3%; *P*=.04), and a higher proportion directed outside the predefined AOIs (mean 36%, SD 16.9% vs mean 55.7%, SD 8.6%; *P*=.02). The unilateral-squat condition also showed a lower proportion of fixation counts directed to the command AOI than the baseline condition (mean 36.2%, SD 8.5% vs mean 44.8%, SD 11.4%; *P*=.04), but it did not show the same increase in outside viewing observed under the concurrent cognitive task condition. Instead, fixation count allocation in the unilateral-squat condition showed small descriptive increases in the score and avatar AOIs.

**Table 3:** Proportion of fixation counts allocated to each area of interest (AOI) across task demand conditions (n=8)^a^.

AOI	Baseline, mean (SD)	Cognitive task, mean (SD)	Unilateral squat, mean (SD)	Cognitive task minus baseline, mean difference (95% CI)	*P* value	Hedges *g*	Unilateral squat minus baseline, mean difference (95% CI)	*P* value	Hedges *g*
Command	44.8 (11.4)	32.1 (8.9)	36.2 (8.5)	−12.7 (−23.7 to −1.7)	.04	−0.86	−8.6 (−17.3 to 0.0)	.04	−0.74
Avatar	5.4 (2.5)	7.4 (6.4)	6.2 (6.8)	2.0 (−3.6 to 7.6)	.66	0.27	0.9 (−3.8 to 5.6)	.92	0.14
Score	14.4 (9.8)	4.9 (4.3)	15.7 (9.0)	−9.5 (−19.9 to 0.9)	.04	−0.68	1.4 (−7.4 to 10.2)	.90	0.12
Outside	36.0 (16.9)	55.7 (8.6)	41.9 (18.4)	19.7 (4.4 to 35.0)	.02	0.95	5.9 (−8.0 to 19.7)	.62	0.32

^a^Baseline: exergame performed in a bilateral stance without an additional task. Cognitive task: exergame performed with serial subtraction during squatting. Unilateral squat: exergame performed in a single-leg stance. Values are presented as mean (SD) of the proportion (%) of fixation counts within each AOI across the entire exergame trial. Proportions were calculated as the number of fixations within each AOI divided by the total number of fixations recorded across the entire exergame trial. Mean differences were calculated as each task demand condition minus the baseline condition. The 95% CIs were calculated from within-participant difference scores. Comparisons with baseline were performed using Dunnett test when parametric assumptions were met; otherwise, paired Wilcoxon signed-rank tests with Holm adjustment were used. For outcomes analyzed with paired Wilcoxon signed-rank tests, *P* values are based on ranked paired differences and may not correspond directly to the mean difference 95% CIs, which are presented descriptively. Effect sizes are reported as Hedges *g* for within-participant comparisons. The “outside” category represents fixation counts allocated outside the 3 predefined AOIs (command, avatar, and score).

### Supportive Analyses

#### Exploratory Correlation Analyses

Correlation analyses between gaze allocation metrics and performance outcomes were conducted on an exploratory basis to generate hypotheses for future studies (Figure S3 in Multimedia Appendix 1). Given the small sample size, these findings should not be interpreted as confirmatory evidence.

#### Descriptive Gaze Distribution

Heat maps and scanpaths are presented as descriptive visualizations to contextualize the quantitative AOI allocation results (Figures S4 and S5 in Multimedia Appendix 1). As these qualitative displays are sensitive to visualization parameters and do not directly quantify gaze allocation, they were not used as stand-alone evidence. Qualitative patterns were interpreted only when consistent with the AOI-based metrics reported in Tables 2 and 3.

## Discussion

### Principal Results

This pilot study suggests that different task demand manipulations within a squat-based exergame may be associated with distinct patterns of gaze redistribution across task-relevant display elements, rather than a uniform reduction in visual attention. Specifically, adding a concurrent cognitive task and performing the task in a unilateral-squat condition produced different descriptive patterns of performance and gaze allocation. By quantifying AOI-specific proportions of fixation time and fixation counts standardized to the entire trial, this approach was intended to reduce potential bias arising from condition-dependent differences in execution time and to generate preliminary, hypothesis-generating estimates for future confirmatory exergame studies.

The manipulation check suggested that the concurrent cognitive task and unilateral-squat conditions affected performance in partially different ways. Specifically, the concurrent cognitive task condition was associated primarily with prolonged execution time while squat performance (score) was largely preserved, whereas the unilateral-squat condition was associated with a more pronounced reduction in squat score together with a modest but significant increase in execution time relative to baseline. Taken together, these patterns suggest that the 2 manipulations may have altered task demands through different mechanisms rather than simply producing a uniform increase in overall difficulty. However, the unilateral-squat condition should not be interpreted as a pure physical load condition because single-leg squatting inherently involves balance control, postural stability, and motor-control processes that may also require cognitive engagement.

The pattern observed in the concurrent cognitive task condition—significantly prolonged execution time, greater fixation allocation outside predefined AOIs, and reduced sampling of the command and score AOIs—is broadly consistent with real-world dual-task evidence showing increased off-task or task-irrelevant fixations when mental arithmetic is superimposed on everyday actions [21,43]. In the present exergame context, this pattern may indicate that the added cognitive task reduced visual sampling of goal-relevant instructional and feedback information while shifting gaze toward non-AOI regions. However, because the Outside category aggregated all fixations outside the 3 predefined AOIs, this interpretation remains tentative. This analysis cannot determine whether these fixations were directed toward peripheral screen areas, visually neutral regions, or other nondefined targets. Therefore, the increase in outside viewing warrants nuanced interpretation. On the one hand, it may reflect weakened top-down attentional selection, whereby limited processing resources reduce the ability to maintain gaze on goal-relevant AOIs. On the other hand, it may reflect strategic gaze aversion. As suggested by Müller et al [21] and Glenberg et al [44], individuals may avert their gaze toward visually neutral or noninformative regions to deliberately reduce external visual input, thereby facilitating demanding internal cognitive processes such as mental arithmetic. In contrast, some laboratory studies using static or tightly controlled stimuli have reported longer fixation durations and fewer fixations under higher cognitive load [45,46], suggesting that gaze responses to cognitive task demands may depend on task dynamics and constraints. One plausible explanation is that squat-based exergaming is both visually dynamic and motorically constrained, requiring intermittent postural-control and feedback sampling, which may promote context-dependent gaze redistribution rather than a single uniform gaze response [47]. Accordingly, these findings are best interpreted as preliminary evidence of context-dependent gaze reallocation during a cognitive dual-task exergame condition rather than as a universal gaze marker of cognitive load [11,20].

By contrast, the unilateral-squat condition showed a different pattern: squat performance decreased significantly, whereas gaze allocation did not show the same increase in outside viewing observed in the concurrent cognitive task condition. Instead, fixation counts directed to the command AOI were reduced, with modest descriptive increases in allocation to other task-relevant AOIs, such as the score and avatar regions. Although these shifts were small and should be interpreted cautiously, they may suggest that unilateral squatting prompted redistribution of visual attention within task-relevant display elements rather than away from them [18,47]. This interpretation is compatible with a tentative self-monitoring explanation, whereby participants may have sampled performance-relevant feedback more frequently as movement difficulty increased. However, this pattern should not be attributed solely to increased physical load because single-leg squatting involves a composite set of demands, including support leg muscular demand, balance control, postural stability, and motor planning. Therefore, the qualitative contrast between the concurrent cognitive task and unilateral-squat conditions should be interpreted as evidence of condition-dependent gaze reallocation under different task demands, not as definitive evidence distinguishing pure cognitive from pure physical load.

These pilot data suggest that gaze metrics may help characterize how users reallocate attention under different task demand conditions during exergaming, which may be useful for hypothesis generation and for identifying candidate features for adaptive systems. However, this study did not evaluate real-time gaze-based adaptation metrics, such as latency, reliability, or within-trial detection, nor did it examine decision thresholds or downstream benefits of adaptation. Therefore, any implications for personalized difficulty adjustment remain speculative and should be reserved for future work.

### Limitations

This study has several limitations. First, the sample was very small and homogeneous, with a marked gender imbalance, which limits generalizability and may influence estimates of multitasking performance and squat execution in some contexts. Replication in a larger, more gender-balanced sample is therefore warranted. Second, statistical power was limited and CIs were often wide. Accordingly, nonsignificant findings should be interpreted cautiously and should not be considered definitive evidence of no effect. The use of proportional AOI metrics is another important limitation. Although normalization by total fixation time or count helped reduce the influence of unequal trial durations across conditions, proportional metrics change the interpretation of the outcome from absolute gaze exposure to relative gaze allocation. Consequently, a decrease in the proportion of fixations directed to a given AOI does not necessarily indicate a decrease in the absolute amount of gaze directed to that AOI. In addition, the AOI-specific findings should be interpreted as exploratory evidence of relative gaze redistribution rather than as independent AOI-specific effects.

Third, the unilateral-squat condition represents a central interpretive constraint of this study. Although single-leg squatting likely increased support leg muscular demand, it cannot be interpreted as a pure physical load manipulation. Single-leg squatting inherently requires balance control, postural stability, motor planning, and continuous movement regulation, all of which may involve cognitive engagement. Therefore, the study design cannot disentangle muscular or physiological load from postural and motor-control demands. In addition, physiological indexes such as heart rate, ratings of perceived exertion, and electromyography were not collected; thus, physiological load differences between conditions could not be directly quantified. Future studies should incorporate objective physiological markers and use designs that can better distinguish muscular effort, postural-control demands, and concurrent cognitive load.

Fourth, the supporting leg in the unilateral-squat condition was fixed to the left side to maintain consistency with the leg sensor and game scoring. This may have introduced unilateral effects because single-leg squat performance can differ between dominant and nondominant limbs. If the left leg was nondominant for some participants, the unilateral-squat condition may have disproportionately increased movement difficulty, potentially contributing to larger decrements in squat score and altered execution time. Such unilateral performance differences could also have secondarily influenced gaze behavior, for example, by increasing monitoring of feedback AOIs such as the score region or by increasing off-AOI viewing, thereby limiting generalizability. Future studies should assess leg dominance and unilateral asymmetries and either counterbalance the supporting leg or include dominance as a covariate.

Finally, standard feasibility indexes such as eye-tracking data loss (eg, fixation coverage) were not systematically quantified in this pilot study. Future studies should prospectively record and report these metrics to better characterize measurement robustness, recruit larger and more diverse cohorts, and examine whether gaze-derived features reliably predict performance or learning at the individual level.

### Conclusions

These pilot data provide preliminary evidence that different task-demand manipulations may be associated with distinct patterns of gaze redistribution during squat-based exergaming in healthy young adults. The findings are hypothesis generating and require replication in larger and more diverse samples. Future studies should determine whether gaze-derived features can be measured robustly at the individual level and translated into validated adaptive difficulty algorithms that improve user outcomes. However, this study does not provide direct evidence for real-time adaptation or clinical utility.

## Appendix

Multimedia Appendix 1 Supplementary figures (Figures S1-S5) showing additional visual materials for the exergame task, area of interest setting, exploratory correlation analyses, and supportive gaze distribution analyses.

## Abbreviations

AOI: area of interest

## Data Availability

The datasets generated or analyzed during this study are not publicly available due to ethical restrictions but are available from the corresponding author on reasonable request.
