# Supplementary material for: Gaze Allocation and Performance Across Task-Demand Conditions During Squat-Based Exergaming: Pilot Study Using Eye Tracking
Source: JMIR Rehabil Assist Technol. 2026 Jun 23;13:e81667. doi: 10.2196/81667 (PMC13342814; doi:10.2196/81667)
Supplement: Multimedia Appendix 1 [file rehab_v13i1e81667_app1.pdf]

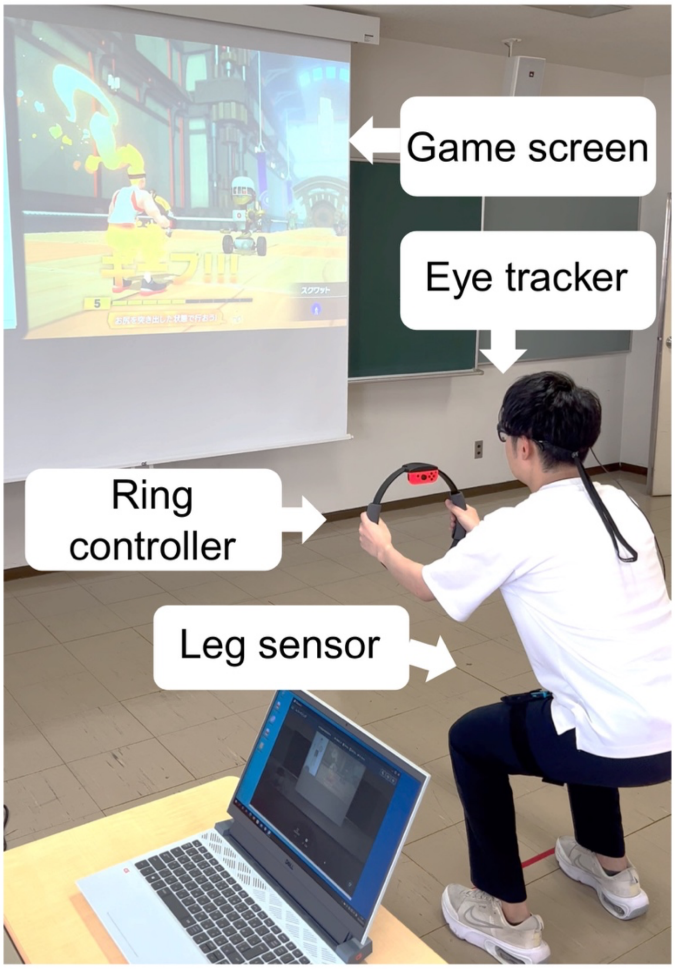

Figure S1. View of the experimental area

The squat-based exergame was performed using Ring Fit Adventure (Nintendo) projected onto a screen positioned approximately at eye level. Participants stood 2.5 m from the screen, wore Tobii Pro Glasses 3 for mobile eye-tracking, and used the Ring-Con with a leg sensor attached to the left thigh. Ambient lighting and surrounding visual conditions were standardized to minimize extraneous visual distractions during data collection.

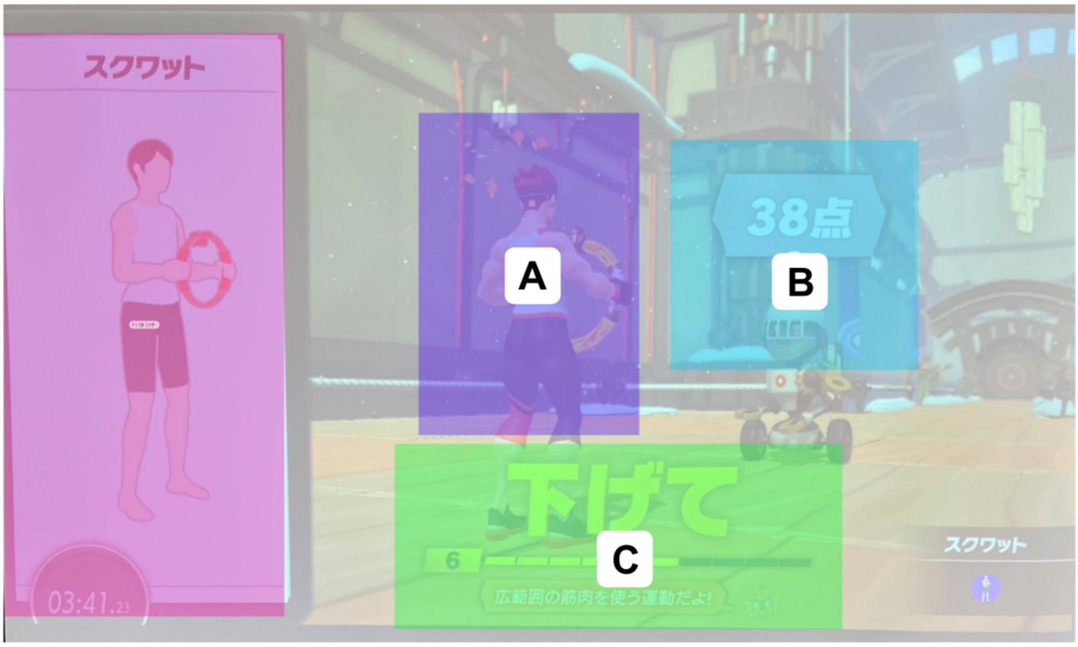

Figure S2. Areas of interest (AOIs) used for eye-tracking analysis in the squat-based exergame.

This figure illustrates the predefined AOIs on the exergame display used for gaze analysis. Panel A shows the Avatar AOI, Panel B the Score AOI, and Panel C the Command AOI.

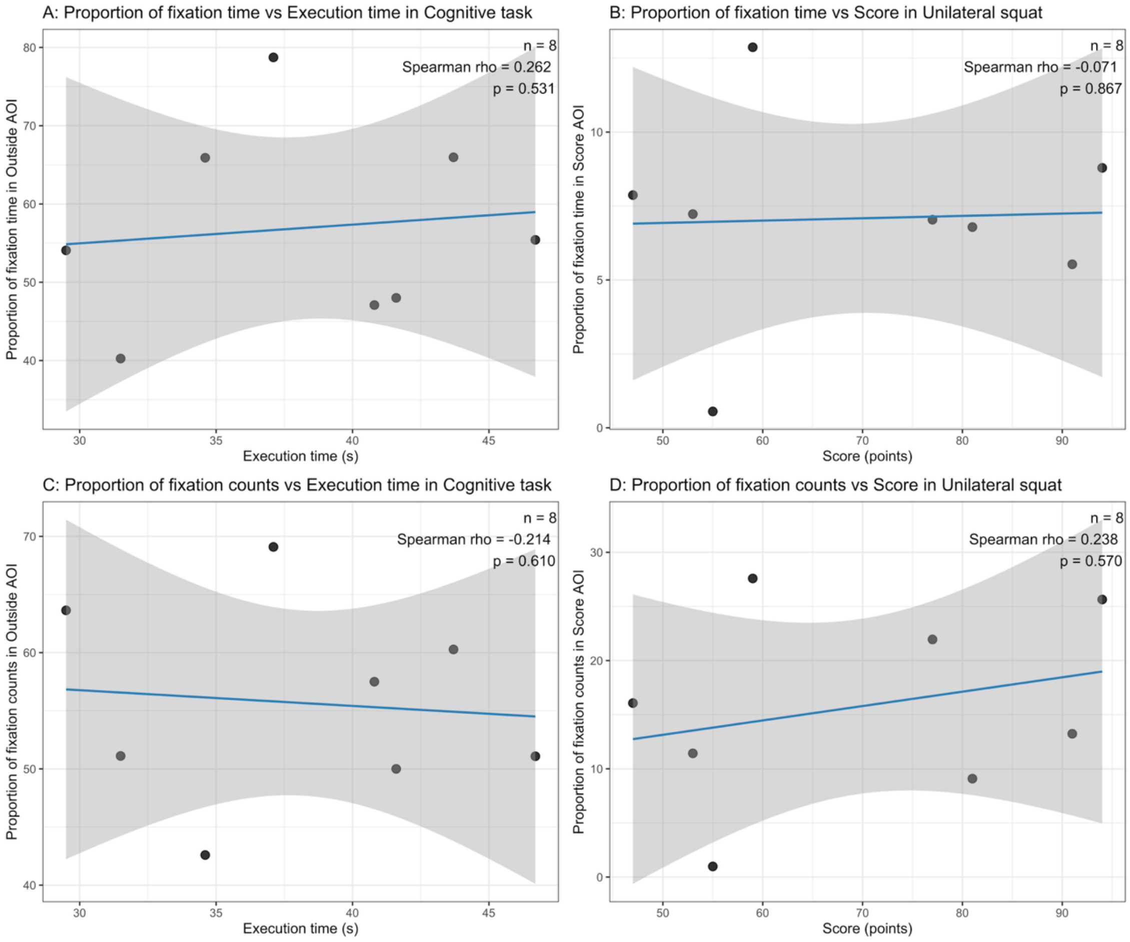

Figure S3. Exploratory correlations between gaze allocation metrics and performance outcomes under concurrent cognitive-task and unilateral squat conditions.

Scatter plots illustrating exploratory associations between gaze allocation metrics and task performance. (A) Proportion of fixation time directed outside predefined areas of interest (AOIs) versus execution time in the concurrent cognitive-task condition. (B) Proportion of fixation time directed to the Score AOI versus squat score in the unilateral squat condition. (C) Proportion of fixation counts directed outside AOIs versus execution time in the concurrent cognitive-task condition. (D) Proportion of fixation counts directed to the Score AOI versus squat score in the unilateral squat condition. Spearman rank correlation coefficients (ρ) and corresponding *p* values are shown in each panel. The blue line represents the fitted regression line with 95% confidence intervals. Analyses were conducted on an exploratory basis to generate hypotheses for future studies and should be interpreted cautiously due to the small sample size ($n = 8$).

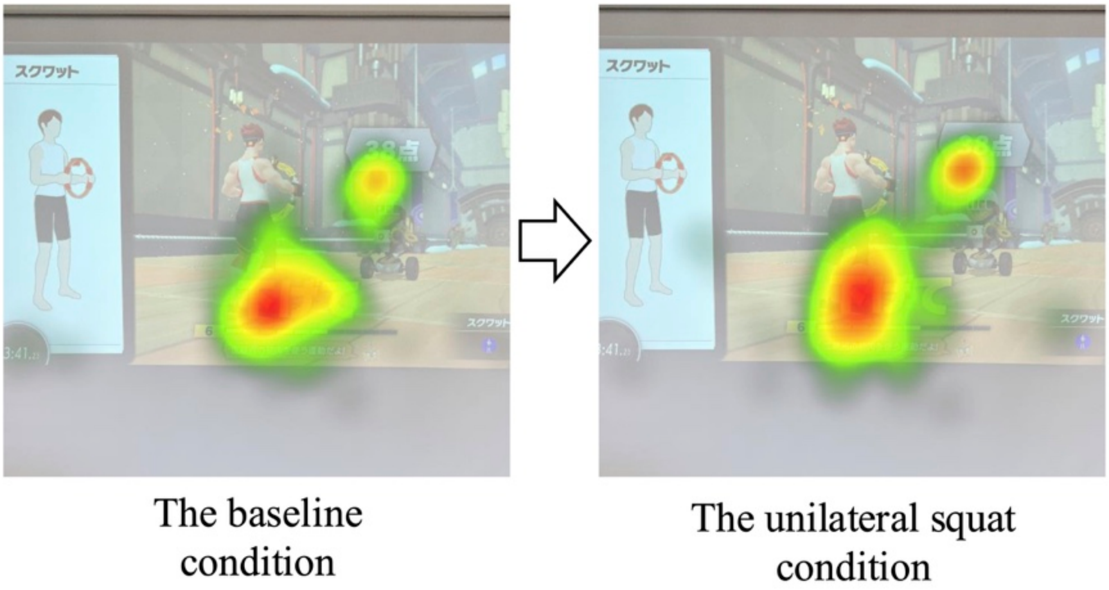

Figure S4. Comparison of gaze heatmaps across experimental conditions.
Heatmaps illustrating the spatial distribution of gaze concentration during squat-based exergame performance under different task conditions. Warmer colors indicate areas with higher gaze density. The baseline and unilateral squat conditions are shown for visual comparison of gaze concentration patterns on the exergame display. These visualizations are provided to qualitatively illustrate differences in gaze allocation across conditions.

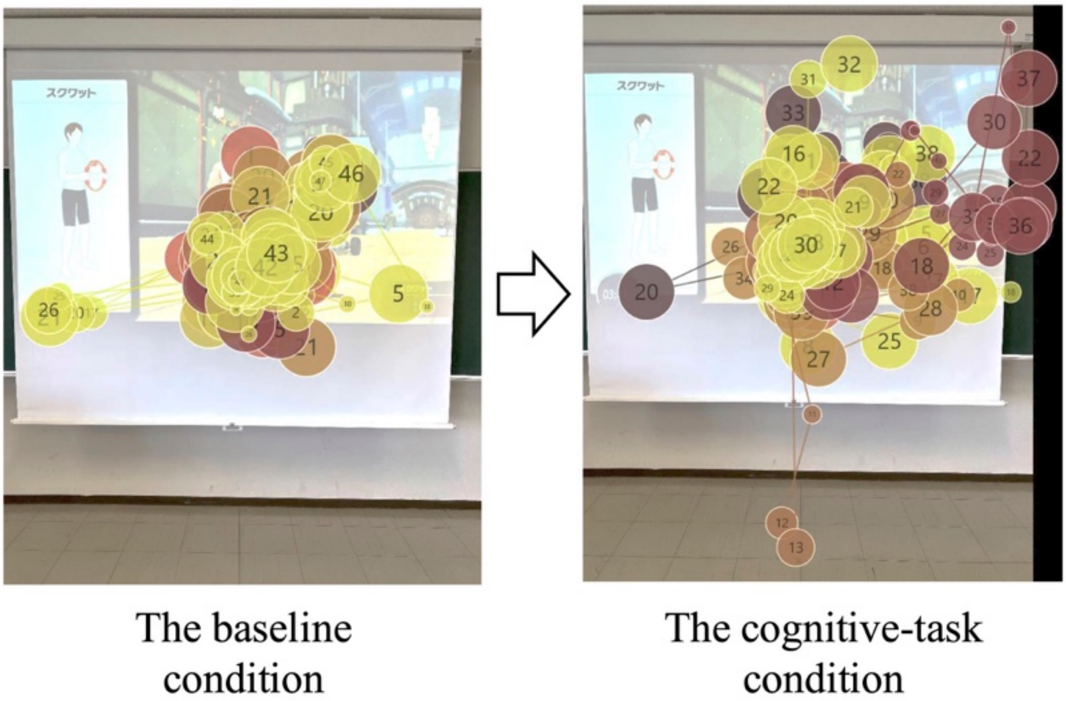

Figure S5. Comparison of scanpaths illustrating eye movement dynamics across conditions.

Representative scanpaths depicting gaze transitions during squat-based exergame performance. The baseline and concurrent cognitive-task conditions are shown to illustrate potential differences in gaze movement patterns under varying task demands. Circles represent fixation locations and lines represent saccadic transitions between fixations.
